# Vitamin D deficiency is not associated with increased oxidative stress in chronic kidney disease pre-dialysis patients

**DOI:** 10.1590/2175-8239-JBN-2019-0156

**Published:** 2020-05-11

**Authors:** Andressa Keiko Matsumoto, Michael Maes, Ana Paula Michelin, Abel Esteves Soares, Laura de Oliveira Semeão, Paula Godeny, Danielle Venturini, Décio Sabbatini Barbosa, Vinicius Daher Alvares Delfino

**Affiliations:** 1 Universidade Estadual de Londrina, Departamento de Patologia, Análises Clínicas e Toxicológicas, Londrina, PR, Brasil.; 2 Deakin University, IMPACT Research Center, Geelong, Australia.; 3 Chulalongkorn University, Faculty of Medicine, Department of Psychiatry, Bangkok, Thailand.; 4 Universidade Estadual de Londrina, Centro de Ciências da Saúde, Programa de Pós-Graduação em Ciências da Saúde, Londrina, PR, Brasil.; 5 Universidade Estadual de Londrina, Departamento de Medicina Interna, Seção de Nefrologia, Londrina, PR, Brasil.

**Keywords:** Renal Insufficiency, Chronic, Oxidative Stress, Vitamin D, Insuficiência Renal Crônica, Estresse oxidativo, Vitamina D

## Abstract

**Introduction:**

The progressive decline in 25-hydroxyvitamin D [25(OH)D] in chronic kidney disease (CKD) limits the kidney ability of synthesizing the vitamin. Vitamin D deficiency as defined by KDIGO (25(OH)D <20 ng/mL) is prevalent in CKD patients and associated to oxidative stress (OS). We studied a possible association between vitamin D deficiency and OS in pre-dialysis patients.

**Methods:**

A cross-sectional study with 206 CKD patients was carried out. Laboratory tests for 25(OH)D, 1,25(OH)2D, inflammatory markers, and OS were added to routine tests including creatinine, albumin, calcium, phosphorus, alkaline phosphatase, iPTH, glucose, hemoglobin, uric acid, total cholesterol, LDL, HDL, and triglycerides.

**Results:**

Vitamin D deficiency was present in 55 CKD patients and normal vitamin D levels were seen in 149 patients. There was a significant association between vitamin D and estimated glomerular filtration rate (eGRF). Homocysteine levels were best predicted by eGRF, sex, and age; high sensitivity C-reactive protein (hsCRP) by staging and BMI; nitric oxide metabolites (NOx) were increased in late disease; leptin was influenced by BMI and higher in women than man; and adiponectin levels were higher in women.

**Conclusions:**

OS biomarkers were not correlated with vitamin D deficiency but increased NOx were seen in stages 4-5 CKD patients. Even though a relatively large number of CKD patients was included and a broad number of OS and inflammatory biomarkers were used in this studied we failed to find an association between vitamin D levels and eGRF. More studies are needed to evaluate the influence of vitamin D status in OS in pre-dialysis CKD patients.

## Resumo

**Introdução:**

A queda da 25-hidroxivitamina D [25 (OH) D] na doença renal crônica (DRC) limita a capacidade renal de sintetizar a vitamina. A deficiência de vitamina D, (25(OH)D<20 ng/mL), é prevalente em pacientes com DRC e associada ao estresse oxidativo (EO). Avaliamos possível associação entre a deficiência de vitamina D e EO em pacientes pré-dialíticos.

**Métodos:**

estudo transversal com 206 pacientes com DRC. Exames para 25(OH)D, 1,25(OH)2D, marcadores inflamatórios e EO foram adicionados àqueles de rotina, incluindo creatinina, albumina, cálcio, fósforo, fosfatase alcalina, iPTH, glicose, hemoglobina, ácido úrico, colesterol total , LDL, HDL e triglicerídeos.

**Resultados:**

55 pacientes com DRC tinham deficiência de vitamina D e os 149 tinham níveis normais da vitamina. Houve uma associação significativa entre a vitamina D e a taxa estimada de filtração glomerular (TFGe). Os níveis de homocisteína foram melhor previstos pela TFGe, gênero e idade; proteína C reativa de alta sensibilidade (hsCRP) por estadiamento e IMC; os metabólitos de óxido nítrico (NOx) aumentaram na doença tardia; a leptina foi influenciada pelo IMC, e mais alta em mulheres, assim como os níveis de adiponectina.

**Conclusões:**

biomarcadores do EO não correlacionaram com a deficiência de vitamina D, mas houve aumento de NOx nos estágios 4-5 da DRC. Apesar dos grandes números de pacientes com DRC, de biomarcadores inflamatórios e EO usados neste estudo, não houve associação entre os níveis de vitamina D e a TFGe. Mais estudos são necessários para avaliar a influência do status da vitamina D no EO em pacientes com DRC em pré-diálise.

## Introduction

In recent years, vitamin D (vit D) is no longer considered to be only a hormone essential to bone metabolism, which deficiency promotes the onset of rickets in children and osteopenia, osteoporosis, and osteomalacia in adults^1^. Its deficiency has been shown to be associated with various clinical conditions, such as secondary hyperparathyroidism, diabetes mellitus, increased cardiovascular risk, and more rapid progression of chronic kidney disease (CKD)^2^.

In clinical practice, serum levels of 25(OH)D are used to define vit D deficiency^1^. In accordance to KDIGO^2^, vitamin D deficiency has been defined as 25(OH)D level <20 ng/mL. Supporting the KDIGO definition, Bouillon et al.^3^ in a critical analysis of evidence-based medicine about optimal vitamin D status confirm the 20 ng/mL cutoff and concluded that serum levels of 25(OH)D above 20 ng/mL are sufficient to normalize calcium and bone homeostasis, as measured by surrogate endpoints such as 1,25(OH)2D, PTHi, calcium absorption, or bone mass.

The prevalence of vit D deficiency is very high in the general population and in CKD patients. In addition, CKD patients have reduced serum levels of the most active form of vit D, 1,25(OH)2D. These deficiencies can be associated with high cardiovascular mortality compared to the general population^4^. Obviously, the high cardiovascular mortality is multifactorial, including traditional cardiovascular (CV) risk factors, as well as uremia-related and non-traditional risk factors such as systemic inflammation and increase oxidative stress (OS), and altered mineral metabolism of bone. Recent evidence suggests that vit D deficiency may contribute to systemic inflammation and increased OS in otherwise healthy people and patients with congestive heart failure^5^.

In certain pathological conditions, including diabetes mellitus, the overproduction of reactive oxygen species (ROS) in the kidneys themselves has been implicated in renal inflammation, glomerular basement membrane derangement, and alteration in tubular function possibly contributing to CKD progression^6^.

Fibroblast growth factor 23 (FGF23) is a key regulator of phosphate and vitamin D metabolism, and it is a known predictor of metabolic bone disease in CKD. The actions of FGF23 include inhibition of 1,25OH2D synthesis and increase in urinary phosphorus. Therefore, excessive serum FGF23 concentrations may interfere with bone mineralization by decreasing the availability of calcium and phosphate substrates^7^. According to Richter et al.^8^, FGF23 and its co-receptor Klotho dysregulations were associated with endothelial dysfunction in humans, but *in vitro* experiments were performed to assess the effects of FGF23 in relation to its co-receptor Klotho on nitric oxide (NO) synthesis and ROS formation via activation of NADPH oxidase 2. In addition ROS degradation via superoxide dismutase 2 (SOD2) and catalase (CAT) is stimulated by FGF23. The authors conclude that FGF23 excess may primarily promote oxidative stress and thus endothelial dysfunction.

It is known that CKD patients have a significant increase of OS and reduced plasma concentrations of 25-dihydroxyvitamin D and 1,25-dihydroxyvitamin D to varying extent^9^. Based upon these studies, we investigated a possible association between deficiency of 25(OH)D and some inflammatory (interleukin 6 [IL-6], leptin, adiponectin, FGF-23 and isoprostane) and oxidative stress (total radical antioxidant parameter [TRAP], lipid hydroperoxide dose [CL-LOOHs], serum concentration of nitric oxide metabolites [NOx], and advanced oxidation protein products [AOPP]) biomarkers in pre-dialysis CKD patients.

## Materials and Methods

### Study protocol

A cross-sectional study was conducted at the CKD clinics of the State University of Londrina and Kidney Institute of Londrina, Brazil, from October 2010 to February 2011. The inclusion criteria were: age greater than 18 years, and CKD stages 3 to 5, according to National Kidney Foundation^10^; all included patients signed the written informed consent for the study. The exclusion criteria were: age less than 18 years, patients that were renal transplant recipients, history of intestinal malabsorption disease, intestinal bypass or bowel resection surgery, presence of liver cirrhosis, infectious disease or active malignancy, unexplained loss of more than 5% of body weight within the last six months, use of steroids orally or via inhalation, use of anticonvulsants drugs, or vit D supplementation. This study was approved by the Ethics Committee of the University of Londrina (086-10 - 07/08/2010), CAAE: 0083.0.268.000-10.

Blood samples (approximately 40 mL) were obtained by venipuncture into vacuum tubes (Vacutainer^®^, Franklin Lakes, NJ USA) after 12 hours of fasting, and were centrifuged for 30 minutes at 3000 rpm (2100 *g*) at 20°C to obtain serum and plasma, and frozen at -80°C.

Vit D deficiency was defined as serum levels of 25(OH) vitamin D (calcidiol or 25(OH)D) <20 ng/mL. Two hundred and sixteen patients met the inclusion criteria and 206 were included in the study. Structured interviews followed by clinical examinations were used to collect data that were relevant to the study objectives, such as gender, age, race / color (self-definition), smoking, presence of hypertension, diabetes, and medications used.

A digital weight scale (Filizola) and a measuring tape were used to measure weight (kg) and height (m), respectively. Body mass indexe (BMI: weight / height^2^), were classified according to the World Health Organization^11^. Waist circumference was measured with a measuring tape at the umbilicus level with patients wearing light clothes, standing with arms at the sides and at the end of expiration, and results were approximated to the nearest centimeter.

### Laboratory tests

Laboratory tests of 25 (OH) D and 1,25(OH) 2D, IL-6, leptin, adiponectin, FGF-23, isoprostane, TRAP, CL-LOOHs, NOx and AOPP were added to routine tests, including creatinine, albumin, calcium, phosphorus, alkaline phosphatase, intact parathyroid hormone (iPTH), glucose, hemoglobin, uric acid, total cholesterol, LDL, HDL, and triglycerides. Routine tests were made soon after blood collection, while the samples for the oxidative stress tests were centrifuged at 3000 rpm (2100 *g*) for 15 minutes (CELM^®^, Barueri, SP, Brazil), and serum obtained was stored in a freezer at -80 °C (70 Indrel^®^ R Londrina, Paraná, Brazil) until use. To determinate the relative protein/creatinine urinary ratio (mg of protein divided by g creatinine) isolated urine samples were used. The inter-assay variability coefficients for all analyses were less than 10%.

The vit D levels were analyzed from serum samples, where the 25(OH)D was measured by chemiluminescence (QL) and 1.25(OH)2D by high efficiency liquid chromatography (HPLC).

### Antioxidant defense and oxidative stress markers

TRAP was evaluated by luminescence in a technical adaptation described by Repetto et al.^12^. Lipid hydroperoxide dose (CL-LOOHs) was measured by an adapted technique described by Flecha et al.^13^. The estimate of nitric oxide metabolites was assessed by determination of serum concentration of NOx by an adaptation of the technique described by Navarro-Gonzalves et al.^14^. To quantify the AOPP, a technique described by Witko-Sarsat et al.^15^ was used. To quantify interleukin 6 (IL-6), leptin, adiponectin, FGF-23, and isoprostane ELISA kits were used (eBioscience, SPI-Bio, SPI-Bio, Cayman Millipore and Eagle Biosciences, respectively).

## Results

### Socio-demographic data

Table 1 shows the baseline socio-demographic and clinical data of the individuals in our study. There were no significant differences in sex ratio, age, ethnicity, BMI, waist circumference, and number of subjects with diabetes, hypertension, obesity, metabolic syndrome (MetS), cardio-vascular events, and use of angiotensin-converting enzyme inhibitors (ACEI), angiotensin receptor blockers (ARB), or statins between subjects with a lower versus normal vit D levels. There was a significant association between the vit D groups and CKD stages.

**Table 1:** Socio-demographic and clinical data of CKD patients of the study according to serum levels of vitamin D.

	Vit D≥20^A^	Vit D<20^B^	F/X^2^	df	p
	(n=149)	(n=55)			
Sex. n. (%)					
Male 68 (33.3%)	44	24	3.60	1	0.058
Female 136 (67.7%)	105	31			
Age (years)	66.3 (13.1)	66.5 (14.3)	0.01	1/202	0.926
Ethnicity – n (%)					
Caucasian - 179 (87.7%)	131	48	0.02	1	0.901
Not Caucasian - 25 (12.3%)	18	7			
CKD Stage. n (%)					
3 (60<GFR >30) - 146 (71.6%)	114B	32	6.85	2	0.033
4 (31<GFR>15) - 49 (24%)	29	20A			
5 (GFR<16) - 9 (4.4%)	6	3			
Diabetic - n (%)					
No 127 (62.3%)	97	30	2.07	1	0.616
Yes 76 (37.3%)	51	25			
Hypertensive - n (%)					
No - 12 (5.9%)	8	4	0.25	1	0.162
Yes - 191 (93.6%)	140	51			
Obesity - n (%)					
No 138 (68.4%)	105	33	1.95	1	0.162
Yes 63 (31.3%)	42	21			
Metabolic Syndrome - n (%)					
No 61 (29.9%)	47	14	0.81	1	0.369
Yes 141 (69.1%)	100	41			
Cardiovascular event - n (%)					
No 121 (59.3%)	89	32	0.04	1	0.842
Yes 83 (40.7%)	60	23			
Use of medicines:					
ACEI - n (%)					
No	108	43	0.68	1	0.410
Yes	41	12			
ARB - n (%)					
No	132	48	0.07	1	0.795
Yes	17	7			
Statin - n (%)					
No	66	18	2.22	1	0.136
Yes	83	37			
BMI (kg/m^2^, median)	28.1 (5.3)	28.9 (5.3)	0.88	1/136	0.348
Waist circuference (cm, median)	103.9 (11.7)	105.5 (10.1)	0.78	1/194	0.378

A significant p value was found between Vit D≥20 ng/mL (A) and Vit D<20 ng/mL (B). CKD: chronic kidney disease; eGFR: estimated glomerular filtration rate; ACEI: angiotensin-converting enzyme inhibitors; ARB: angiotensin receptor blockers; BMI: body mass index

### Associations between vitamin D and metabolic variables

Table 2 shows the differences in the metabolic variables between individuals with low vit D levels versus those with normal vit D levels. It shows decreased 25(OH)D (F=176.47, df=1/202, p=<0.001), albumin (F=5.81, df=1/201, p=0.017), eGFR (F=6.83, df=1/202, p= 0.010) in the subjects with a lower versus normal vit D levels. In addition, concentration of alkaline phosphatase (F=5.29, df=1/201, p=0.022), iPTH (F=10.99, df=1/201, p=0.001), total cholesterol (F=7.81, df=1/202, p=0.006), LDL (F=4.02, df=1/196, p=0.046), HDL (F=4.02, df=1/196, p=0.046), urinary protein/creatinine ratio (F=16.02, df=1/195, p=0.001) were higher in CKD patients with vit D deficiency.

**Table 2:** Laboratory and eGFR data in participants according to serum levels of vitamin D.

	Vit D≥20 ng/mL	Vit D<20 ng/mL	F	df	p
	(n=149)	(n=55)			
25(OH)D (ng/mL)	35.1 (11.1)	14.6 (4.0)	176.47	1/202	<0.001
1.25(OH)_2_D (pg/mL)*	29.0 (9.0)	86.2 (6.6)	3.32	1/202	0.070
FGF-23* (pg/mL)	28.6 (31.7)	39.2 (70.0)	0.13	1/200	0.718
Calcium (mg/dL)*	8.8 (3.3)	8.3 (0.7)	2.53	1/201	0.114
Phosphorus (mg/dL)	3.6 (0.7)	3.8 (0.71)	3.20	1/202	0.075
Alkaline Phosphatase (U/L)*	97.2 (34.3)	111.4 (44.0)	5.29	1/201	0.022
iPTH (pg/mL)*	139.3 (104.2)	188.3 (103.9)	10.99	1/201	0.001
Albumin (g/dL)*	4.35 (3.19)	3.88 (0.61)	5.81	1/201	0.017
Glucose (mg/dL)	110.6 (31.1)	112.7 (47.1)	0.05	1/201	0.818
Uric acid (mg/dL)	7.29 (3.46)	6.94 (2.00)	3.69	1/201	0.818
Total cholesterol (mg/dL)	173.2 (43.5)	192.6 (44.4.)	7.81	1/202	0.006
LDL (mg/dL)	102.2 (34.3)	114.0 (42.0)	4.02	1/196	0.046
HDL (mg/dL)	43.0 (11.0)	45.6 (12.9)	4.02	1/196	0.046
Triglycerides (mg/dL)*	149.3 (101.4)	152.2 (83.3)	0.11	1/202	0.746
Creatinine (mg/dL)	1.95 (0.80)	2.16 (0.92)	3.07	1/202	0.081
eGFR (mL/min/1.73 m^2^)	37.2 (12.3)	32.3 (11.1)	6.83	1/202	0.010
Ratio urinary Protein/creatinine (mg/g)*	0.51 (0.81)	1.55 (2.60)	16.02	1/195	0.001

All results are shown as mean (±SD).

* Variables are processed in Ln transformation. Analyses of variance was used to compare groups. FGF-23: Fibroblast Growth Factor; iPTH: intact parathyroid hormone, LDL: low density lipoprotein; HDL: high density lipoprotein; eGFR: estimate glomerular filtration rate.

Therefore, we have carried out a multivariate analysis (Table 3) with the 16 metabolic markers as dependent variable and 25(OH)D (F=3, df=16/159, p<0.001) as explanatory variables. If this multivariate GLM analysis showed a significant effect of the explanatory variables, we further explored the associations using between-subject analyses. The univariate analysis showed that eGFR (F=6.29, df=1/174, p=0.013) and Ln albumin (F=13.53, df=1/174, p<0.001) were positively associated with 25(OH)D and the variables urea (F= 5.83, df=1/174, p=0.017), Ln creatinine (F=4.52, df=1/174, p=0.035), urinary prot/creat ratio (F=24.75, df=1/174, p<0.001), and total cholesterol (F=4.39, df=1/174, p=0.038) were negatively associated with 25(OH)D.

**Table 3:** Effects of Vit D concentrations on the 16 metabolic variables listed in Table 2.

Analyses	Dependent	Exploratory	F	df	p
	variables	Variables			
Multivariate	All 16 variables	Vit 25OH(+)*	3.00	16/159	<0.001
Univariate	eGFR	Vit 25OH (+)	6.29	1/174	0.013
	Urea	Vit 25OH (-)	5.83	1/174	0.017
	Ln creatinine	Vit 25OH (-)	4.52	1/174	0.035
	Ln albumin	Vit 25OH (+)	13.53	1/174	<0.001
	Ratio urinary prot/creat	Vit 25OH (-)	24.75	1/174	<0.001
	Total cholesterol	Vit 25OH (-)	4.39	1/174	0.038

* The effects of stage, sex, age, and BMI on the dependent variables are shown in Table 2. Vit 25OH (+): normal levels of vit D; Vit 25OH (-): deficiency of vit D. The analyses were done after controlling for the effect of diabetes (F=4.82, df=16/158, p<0.001).

### Effects of unrelated and confounding variables

We also controlled for possible effects of other putative intervening variables (listed in Table 1). Thus, after controlling for effects of ethnicity (F=3.68, df=16/158, p<0.001; ethnicity had significant effects on eGFR and LDL-cholesterol), the effects of vit D groups on the 16 markers remained significant (F=3.04, df=16/158, p<0.001). After controlling for effects of the MetS (F=6.26, df=16/157, p<0.001) significant effects of vit D deficiency were found on concentration of phosphorous, albumin, HDL-cholesterol and triglycerides. (F=2.99, df=16/157, p<0.001). In univariate analysis, even after controlling for effects of diabetes, significant effects on urea, phosphorous, glucose, and urinary protein/creatinine ratio were found (F=3.03, df=16/158, p<0.001). In multivariate GLM analyses, serum levels of vit D influenced the 16 markers studied (F=3.00, df=16/159, p=<0.001)

### Associations of vit D and IO&NS variables

Table 4 presents the inflammation, oxidative & nitrosative stress (IO&NS) variables in subjects with low versus normal vit D values. This table shows the results of ANOVAs for the 10 variables although it is more appropriate to evaluate these differences using Table 5, after adjusting for confounding variables. Table 5 shows that using multivariate GLM analysis there was no significant effect of the vit D groups on the 10 IO&NS markers, whereas eGFR staging, sex, age, and BMI had all significant effects. Homocysteine was best predicted by eGFR staging, sex and age, hsCRP by staging and BMI, NOx by staging, leptin by sex, and BMI and adiponectin by sex.

**Table 4:** Measurements of inflammatory and oxidative stress variables in participants according to their vitamin D concentrations.

Variables	≥20 ng/mL	<20 ng/mL	F	df	p
Leptin (ng/mL)	38.0 (39.6)	41.4 (35.9)	1.48	1/202	0.225
Adiponectin (ng/mL)	21.1 (10.9)	23.1 (11.2)	1.50	1/202	0.230
hsCRP (mg/dL)	6.9 (18.1)	12.6 (50.9)	2.46	1/196	0.118
NOx (µM/L)	8.59 (5.71)	8.36 (4.24)	0.74	1/202	0.785
IL-6 (pg/mL)	8.3 (22.4)	5.5 (6.2)	0.007	1/201	0.933
F2- Isoprostane (pg/mL)	160.4 (258.7)	193.0 (379.8)	2.14	1/201	0.145
AOPP (µmol/L)	103.4 (31.7)	111.7 (41.3)	1.80	1/202	0.181
Hydroxyperoxide (cpm)	42727.5 (27858.6)	42685.4 (31295.3)	0.30	1/202	0.863
TRAP/UA (µmol trolox/mg/dL)	132.7 (30.7)	139.4 (38.9)	1.62	1/201	0.204
Homocysteine (µmol/L)	24.0 (8.4)	24.2 (8.4)	0.02	1/198	0.877

CRP: C-reactive protein; IL-6: Interleukin-6; AOPP: advanced oxidation protein products; TRAP/UI: total radical antioxidant parameter/uric acid.

**Table 5:** Results of multivariate GLM analysis with the 10 inflammatory/oxidative stress (IO&NS) biomarkers as dependent variables and vitamin D groups, stage, sex, age, and body mass index (BMI) as explanatory variables.

Analyses	Dependent	Explanatory	F	df	p
	variables	variables			
Multivariate	All 10 IO&NS variables	Vit D groups (low vs normal)	0.62	10/169	0.792
		Stages (3/4/5)	2.25	20/340	0.002
		Sex	12.31	10/169	<0.001
		Age	1.89	10/169	0.049
		BMI	9.10	10/169	<0.001
Univariate	Homocysteine	Stage (4+5>3)	11.00	2/178	<0.001
		Sex (M>F)	8.73	1/178	0.004
		Age (+)	5.70	1/178	0.018
	CRP	Stage (4+5>3)	4.78	2/178	0.009
		BMI (+)	11.59	1/178	0.001
	NOx	Stage (4+5>3)	6.30	1/178	0.002
	Leptin	Sex (F>M)	67.39	1/178	<0.001
		BMI (+)	79.49	1/178	<0.001
	Adiponectin	Sex (F>M)	22.30	1/178	<0.001

CRP: C-reactive protein; NOx: nitric oxide synthase.

## Discussion

Our findings complement observations suggesting that 25(OH)D levels are positively associated with eGFR levels and alkaline phosphatase, and negatively with urea, creatinine, urinary protein/creatinine ratio, and total cholesterol. It is known that the majority of CKD patients have restricted protein and caloric intake, which contributed to the relatively low vit D levels seen in this population. Besides, many CKD patients have limited outdoor physical activities, with reduced exposure to sunlight, and greater loss of urinary vit D metabolites occurs in CKD patients with overt proteinuria, factors that also contribute to vit D deficiency.

A Brazilian study investigated more than 1.800 CKD patients in various stages of the disease, with 25(OH)D deficiency (<22.0 pg/mL) in more than 60% of those with eGFR <30 mL/min/1.73m^2^ 16.

Prevalence of 25(OH)D deficiency is common in CKD and it is implicated in PTH progressive increase that is seen with declining renal function and leads to secondary hyperparathyroidism, mineral bone disease, and increased cardiovascular risk^17^.

Albuminuria, increased urinary protein/creatinine ratio, and low 25(OH)D level are typical findings in CKD patients^18^. According to Oh et al.^19^, LDL and total cholesterol levels were higher in CKD patients with vit D deficiency than the control group.

Our data did not corroborate the findings of Milovanova et al.^20^ studies that showed lower FGF-23 in CKD 3b-4 stage patients.

Adipose tissue has been recognizing as an endocrine tissue releasing various factors involved in vascular functions, energy metabolism, or inflammation. Among them are interleukins such IL-6, tumor necrosis factor alpha (TNF-α), leptin, and adiponectin. In humans, hyperleptinemia has been shown to be associated with CKD progression^21^. Plasma levels of adiponectin are higher in CKD patients and correlate positively with CKD stage and albuminuria^22^.

It has been shown that C-reactive protein (CRP) concentrations seems to be associated with later stages of CKD and leptin, and adiponectin with BMI and sex. Numerous studies have reported an inverse association between renal impairment level and different mediators and biomarkers of inflammation including CRP, IL-6, TNF-α, and fibrinogen, pointing to a high prevalence of increased inflammation in CKD^23^.

The increased adiponectin, leptin, and IL-6 and dysregulation of 1.25(OH)2D are associated with increased FGF23 levels, and vit D deficiency is associated with low leptin. Notably, adiponectin levels are elevated by the degree of renal insufficiency^24^.

Rutkowski et al.^25^ described that adiponectin primarily affected renal α-klotho expression and bone FGF23 release; high adiponectin levels suppress renal α-klotho secretion, reduce plasma FGF23 levels, and cause renal loss of calcium.

A 2017 study reported lower serum adiponectin levels in patients with type 2 diabetes mellitus (T2DM) and stage 1 CKD than in patients with T2DM without renal pathology, with progressively higher levels coinciding with the deterioration of kidney function^26^.

According to Agarwal et al.^27^, OS has an important role in CKD disease; the biologic markers of OS are found increased in cell cultures, animal models, and end-stage renal disease patients on hemodialysis.

Kidney cells contain abundant mitochondria and therefore their dysfunction have a fundamental role in the progression of renal failure. Many reports point out to a role of mitochondria in the increased OS in kidney disease 28 ^,^ 29. Deposition of uric acid crystals in proximal tubule cells is also associated with increased OS and activation of the inflammatory system^30^.

According to Dounousi et al.^31^, levels of different markers including plasma F2-isoprostanes, advanced oxidation protein products, and malondialdehyde are increased in patients with varying degrees of renal function, including patients with end-stage renal failure.

The major finding of this study was that no association was found between vitamin D deficiency and increased state redox in CKD patients. The OS biomarkers (NOx) was not associated with vit D deficiency, but increased NOx levels were significantly higher in more advanced stages of CKD. Although many studies have demonstrated that vit D deficiency is associated with inflammation and OS in CKD patients, recent studies failed to show this association 32 ^,^ 33.

Our data showed an increased NOx in stages 4 and 5 CKD patients. It is known that NO is synthesized in several cell types, being the vascular endothelium the major source of NO synthesis. The measurement of serum NOx levels estimates basal NO generation by endothelial cells. Therefore, it may be speculated that increased serum NOx in CKD people may be due to endothelial NOS (eNOS) inhibition and inducible NOS (iNOS) overexpression^34^. In most cell types, including adipocytes, iNOS is induced by inflammatory signals when hypoxia favors cell necrosis, which is a process that recruits macrophages and other phagocytic cells, and creates a milieu favoring the perpetuation of inflammation within the adipose tissue^35^. Moreover, when superoxide anion and NO are produced simultaneously in close proximity, a reaction leads to the formation of peroxynitrite (ONOO^-^), and, subsequently, to hydroxyl radicals, amplifying the oxidative process on biomolecules^36^.

Uncoupling of eNOS and defective production of NO results in impaired vasorelaxation of renal resistance arteries, diapedesis of polymorphonuclear leukocytes and monocytes, and local procoagulant and a proaggregant conditions. Local vasoconstriction is further exaggerated by the loss of endothelial integrity. These vascular events are followed by tubular mechanisms that include not only the induction of iNOS and increased production of reactive oxygen intermediates in the renal epithelial cells, but also generation of ONOO^-^ by the infiltrating polymorphonuclear cells and macrophages^37^.

Some of the deleterious effects induced by hyperuricemia are similar to those associated with increased OS such as reduced NO bioavailability and endothelial dysfunction, vascular hypertrophy, and inflammation^38^. However, our findings did not reveal differences between the groups with and without vit D deficiency. The inhibitory influences include increase in the endogenous NOS inhibitors such as asymmetric dimethylarginine (ADMA) and decrease in activity of the NOS enzymes for many reasons, including the reduction in protein abundance events that reduce inherent enzyme activity and reduced availability of essential cofactors. In addition, deposition of advanced glycosylated end-products occurs in advanced renal disease, which decreases the access of NO to its target tissue and contributes to NO deficiency 39 ^,^ 40.

Our study has limitations, such as the small number of stage 5 CKD, limiting our interpretation about the relationship of vit D and OS in that group. In addition, this was a single-center cross-sectional study, not allowing conclusion on the causality between vit D deficiency and redox state. Even with these limitations, we believe that our results may be reliable, as we included a relatively large number of patients and used a broad panel of OS biomarkers.

## Conclusion

Our findings suggested that vit D deficiency may not play a very important role in the increased OS state seen in pre-dialysis CKD patients, which is clearly multifactorial. Due to the cross-sectional study design, we were unable to exclude that vit D supplements could ameliorate OS in similar patients, question that could only be answered by an adequately powered clinical trial.
